# The impact of rapid molecular diagnostic testing for respiratory viruses on outcomes for emergency department patients

**DOI:** 10.5694/mja2.50049

**Published:** 2019-03-05

**Authors:** Nasir Wabe, Ling Li, Robert Lindeman, Ruth Yimsung, Maria R Dahm, Kate Clezy, Susan McLennan, Johanna Westbrook, Andrew Georgiou

**Affiliations:** ^1^ Centre for Health Systems and Safety Research Australian Institute of Health Innovation Macquarie University Sydney NSW; ^2^ NSW Health Pathology Sydney NSW; ^3^ Prince of Wales Hospital Sydney NSW; ^4^ Sydney Medical School University of Sydney Sydney NSW

**Keywords:** Emergency services, medical, Public health, Diagnostic tests and procedures

## Abstract

**Objective:**

To determine whether rapid polymerase chain reaction (PCR) testing for influenza and respiratory syncytial viruses (RSV) in emergency departments (EDs) is associated with better patient and laboratory outcomes than standard multiplex PCR testing.

**Design, setting:**

A before‐and‐after study in four metropolitan EDs in New South Wales.

**Participants:**

1491 consecutive patients tested by standard multiplex PCR during July–December 2016, and 2250 tested by rapid PCR during July–December 2017.

**Main outcome measures:**

Hospital admissions; ED length of stay (LOS); test turnaround time; patient receiving test result before leaving the ED; ordering of other laboratory tests.

**Results:**

Compared with those tested by standard PCR, fewer patients tested by rapid PCR were admitted to hospital (73.3% *v* 77.7%; *P* < 0.001) and more received their test results before leaving the ED (67.4% *v* 1.3%; *P* < 0.001); the median test turnaround time was also shorter (2.4 h [IQR, 1.6–3.9 h] *v* 26.7 h [IQR, 21.2–37.8 h]). The proportion of patients admitted to hospital was also lower in the rapid PCR group for both children under 18 (50.6% *v* 66.6%; *P* < 0.001) and patients over 60 years of age (84.3% *v* 91.8%; *P* < 0.001). Significantly fewer blood culture, blood gas, sputum culture, and respiratory bacterial and viral serology tests were ordered for patients tested by rapid PCR. ED LOS was similar for the rapid (7.4 h; IQR, 5.0–12.9 h) and standard PCR groups (6.5 h; IQR, 4.2–11.9 h; *P* = 0.27).

**Conclusion:**

Rapid PCR testing of ED patients for influenza virus and RSV was associated with better outcomes on a range of indicators, suggesting benefits for patients and the health care system. A formal cost–benefit analysis should be undertaken.

**The known**: Rapid polymerase chain reaction (PCR) testing for influenza and respiratory syncytial viruses (RSV) was introduced in New South Wales in July 2017. Its impact on outcomes for emergency department (ED) patients has not been investigated.

**The new**: Compared with standard PCR testing, rapid PCR was associated with significantly fewer hospital admissions, more rapid test turnaround, more patients receiving test results before leaving the ED, and reduced numbers of some other common microbiology tests. It did not significantly affect ED length of stay.

**The implications**: Rapid PCR testing of ED patients for major respiratory viruses can benefit patients and reduce resource use.

The health and economic burdens associated with acute respiratory infections by influenza and respiratory syncytial viruses (RSV) are significant in Australia and overseas.^1^, ^2^, ^3^ Polymerase chain reaction (PCR) testing is effective for confirming respiratory viral infections.^4^ Multiplex PCR can detect numerous respiratory viruses, including influenza and parainfluenza viruses, RSV, adenovirus, rhinovirus, human metapneumovirus, enterovirus, bocavirus and coronavirus with very high sensitivity and specificity.^5^ Although the results of standard multiplex PCR are accurate and comprehensive, it has traditionally been performed in a central laboratory with a lengthy turnaround time, which may be inconvenient in settings where test results are urgently required, including emergency departments (EDs).

Rapid, easy‐to‐use PCR‐based respiratory virus diagnostic tests have been introduced in recent years;^6^, ^7^ the GeneXpert system (Cepheid), for instance, was introduced in New South Wales in July 2017. Rapid PCR tests were expected to facilitate timely and appropriate initiation of treatment, improve outbreak prevention and infection control measures, and expedite the assessment of patients in EDs.

In this study, we analysed routinely collected data to determine whether rapid PCR testing for influenza and RSV infections in EDs is associated with improved patient and laboratory outcomes. We compared data for patients tested for influenza A/B viruses and RSV immediately after rapid PCR diagnosis was introduced (July–December 2017) with data for patients tested with a standard multiplex PCR system during July–December 2016.

## Methods

### Setting

We undertook a before‐and‐after study in four metropolitan public hospital EDs in Sydney, NSW: three general hospitals (EDs A, B and C; 76 228, 54 443 and 50 025 annual ED presentations respectively) and one children's hospital (ED D; 36 700 annual ED presentations; all data for January–December 2016^8^). The four hospitals were served by a single pathology laboratory provider.

### Populations and data sources

We analysed data for all patients tested for influenza virus or RSV. During July–December 2016, patients were tested with the standard PCR system, a central laboratory‐based multiplex PCR test for sixteen respiratory viruses (including RSV and influenza viruses A and B), available as a referral test at the central laboratory in Hospital B. During July–December 2017, patients were tested with the rapid PCR system, a hospital laboratory‐based test specific for RSV and influenza viruses A and B. Hospitals A, B and D have onsite laboratories that perform rapid PCR testing; Hospital C sends samples to the nearby Hospital A.

All tests were conducted in virology laboratories by trained staff, and test results were entered into laboratory information system datasets. We obtained relevant patient characteristics and laboratory data by linking the ED and laboratory information system datasets. Detailed information about the datasets and the linkage process have been described previously.^9^

### Outcomes

The primary outcomes were admission to hospital, ED length of stay (LOS), test turnaround time, and the patient receiving their test result before leaving the ED. ED LOS was defined as the time from arrival in the ED to discharge or admission to hospital. Turnaround was defined as the time from when the sample was received by the laboratory to when the test result was available in hospital electronic records. As secondary outcomes, we compared ordering of typical biochemistry and haematology tests (full blood count; electrolyte, urea, creatinine levels; liver function test; blood gas analysis; C‐reactive protein level) and microbiology tests (blood culture; urine microscopy, culture and sensitivity analysis; sputum culture; respiratory bacteria and virus serology) during the two study periods.

### Statistical analysis

Analyses were conducted in Stata 15 (StataCorp). Descriptive statistics are reported (medians with interquartile ranges [IQRs], means with standard deviations [SDs], numbers with proportions). Baseline characteristics were compared in *χ* ^2^ tests (categorical outcomes) or Wilcoxon rank‐sum tests (continuous outcomes). Logistic regression analyses of binary outcomes (eg, hospital admission: yes/no) and quantile regression analyses of continuous outcomes (eg, ED LOS) were undertaken. All analyses were adjusted for baseline characteristics.

Sensitivity analyses limited to data for children (under 18 years of age) or older patients (over 60 years of age) were conducted. *P* < 0.05 (2‐tailed) was deemed statistically significant.

### Ethics approval

Ethics approval for the investigation was granted by the Human Research Ethics Committee of the South Eastern Sydney Local Health District (reference, HREC/16/POWH/412).

## Results

### Baseline characteristics and PCR test results

We analysed data for 3741 patients presenting to the four EDs during two periods: 1491 consecutive patients during July–December 2016 (standard PCR) and 2250 during July–December 2017 (rapid PCR). Baseline characteristics for the two groups were similar in terms of sex, triage category, and arrival day of the week, but differed significantly for age, arrival time, and mode of arrival (Box 1). Among those tested by rapid PCR, supplementary standard PCR tests were ordered for 133 patients (5.9%), and standard PCR tests for respiratory viruses other than influenza viruses A/B and RSV were requested for a further 320 patients (14.2%).

**Box 1:** Baseline characteristics of 3741 patients who presented to four Sydney metropolitan emergency departments (EDs) and were tested for influenza and respiratory syncytial viruses with standard or rapid polymerase chain reaction (PCR) systems

Characteristics	Standard PCR (July–Dec 2016)	Rapid PCR (July–Dec 2017)	*P*
Number of patients	1491	2250	
Sex (male)	759 (50.9%)	1103 (49.0%)	0.26
Age (years), median (IQR)	53 (6–77)	69 (41–82)	< 0.001
Triage category			0.68
Resuscitation	33 (2.2%)	47 (2.1%)	
Emergency	352 (23.6%)	567 (25.2%)	
Urgent	902 (60.5%)	1314 (58.4%)	
Semi‐urgent	198 (13.3%)	309 (13.7%)	
Non‐urgent	6 (0.4%)	13 (0.6%)	
ED arrival: time			< 0.001
7 pm–7 am	571 (38.3%)	688 (30.6%)	
7 am–7 pm	920 (61.7%)	1562 (69.4%)	
ED arrival: day of week			0.16
Weekday	1082 (72.6%)	1679 (74.6%)	
Weekend	409 (27.4%)	571 (25.4%)	
Mode of arrival			< 0.001
Private/public transport	920 (61.7%)	1154 (51.3%)	
Ambulance/other	571 (38.3%)	1096 (48.7%)	
Emergency department			< 0.001
A	517 (34.7%)	1173 (52.1%)	
B	473 (31.7%)	371 (16.5%)	
C	199 (13.4%)	553 (24.6%)	
D	302 (20.2%)	153 (6.8%)	

IQR = interquartile range. ◆

A total of 134 people in the standard PCR group (9.0%) were positive for influenza A/B (37 patients), RSV (96 patients), or both (one patient); a further 333 people (22.3%) were positive for at least one other respiratory virus (but not influenza A/B or RSV). Of the patients in the rapid PCR group, 790 (35.1%) were positive for influenza A/B (732 patients), RSV (56 patients), or both (two patients). In the children's hospital (ED D), 53 children in the standard PCR group (18%) were positive for RSV (49 children) or influenza A/B (five children); a further 144 (48%) were positive for at least one other respiratory virus. Of those tested by rapid PCR, 54 children (35%) were positive for influenza A/B (46 children) or RSV (eight children).

### Primary outcomes

A smaller proportion of patients tested by rapid PCR were admitted to hospital (73.3% *v* 77.7%; *P* < 0.001 after controlling for baseline characteristics) and a larger proportion received their test result before leaving the ED (67.4% *v* 1.3%, *P* < 0.001) than of patients tested by standard PCR. The median turnaround for rapid PCR tests was shorter (rapid PCR, 2.4 h [IQR, 1.6–3.9 h]; normal PCR, 26.7 h [IQR, 21.2–37.8 h]), but the median ED LOS was similar for the two groups (rapid PCR, 7.4 h [IQR, 5.0–12.9 h]; normal PCR, 6.5 h [IQR, 4.2–11.9 h]; *P* = 0.27) (Box 2).

**Box 2:** Primary outcomes for 3741 patients at four metropolitan emergency departments (EDs) tested for influenza and respiratory syncytial viruses by standard or rapid polymerase chain reaction (PCR)

Outcomes	Standard PCR	Rapid PCR	Adjusted odds ratio* (95% CI)	*P*
Number of patients	1491	2250		
Admitted to hospital	1159 (77.7%)	1649 (73.3%)	1.9 (1.6–2.3)	< 0.001
ED length of stay (h), median (IQR)	6.5 (4.2–11.9)	7.4 (5.0–12.9)	—	0.27
Test turnaround time (h), median (IQR)	26.7 (21.2–37.8)	2.4 (1.6–3.9)	—	< 0.001
Patients received test result in ED	19 (1.3%)	1516 (67.4%)	0.006 (0.004–0.009)	< 0.001

CI = confidence interval; IQR = interquartile range. ^*^ Standard PCR *v* rapid PCR; adjusted for baseline characteristics (Box 1). ◆

Among patients with positive test results, 105 patients tested by standard PCR (78.4%; 95% CI, 70.4–85.0%) and 532 patients tested by rapid PCR (67.3%; 95% CI, 63.9–78.6%) were admitted to hospital (after adjusting for baseline characteristics: adjusted odds ratio [aOR], 4.0; 95% CI, 2.3–6.8). For patients with negative results, 1054 people tested by standard PCR (77.7%; 95% CI, 75.4–79.9%) and 1117 of those tested by rapid PCR (76.5%; 95% CI, 74.2–78.7%) were admitted to hospital (after adjusting for baseline characteristics: aOR, 1.5; 95% CI, 1.2–1.8) (Box 3).

**Box 3:**
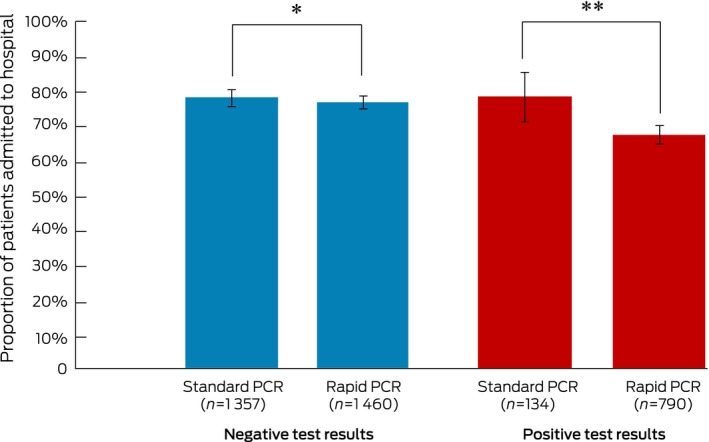
Hospital admission rates (with 95% confidence intervals) for 3741 patients at four Sydney metropolitan emergency departments (EDs) tested for influenza and respiratory syncytial viruses by standard or rapid polymerase chain reaction (PCR), by test result After adjusting for baseline characteristics (Box 1): * *P* = 0.012; ** *P* < 0.001. ◆

### Secondary outcomes

The overall numbers of tests per patient were similar in the standard PCR (mean, 7.2 tests; SD, 3.8) and rapid PCR groups (mean, 7.1 tests; SD, 3.4). The mean number of microbiology tests per patient was significantly lower for the rapid PCR group (1.5 tests; SD, 1.8) than for the standard PCR group (2.0 tests; SD, 2.1; *P* < 0.001 after controlling for baseline characteristics).

The 16 265 biochemistry/haematology and microbiology tests comprised 71.1% of the 22 876 other tests (that is, not including PCR virus testing) ordered for patients in the study. After adjusting for baseline characteristics, the proportions of patients for whom full blood count, electrolyte/urea/creatinine levels, liver function, or C‐reactive protein were assessed were similar, as were the proportions for urine microscopy, culture and sensitivity tests. Significantly fewer blood culture, blood gas, sputum culture, and respiratory bacterial and viral serology tests were ordered for patients in the rapid PCR group (Box 4).

**Box 4:** Secondary outcomes for 3741 patients at four Sydney metropolitan emergency departments (EDs) tested for influenza and respiratory syncytial viruses by standard or rapid polymerase chain reaction (PCR): other laboratory test ordering

Test ordered	Standard PCR	Rapid PCR	Adjusted odds ratio* (95% CI)	*P*
Total number of patients	1491	2250		
**Biochemistry/haematology**				
Full blood count	1261 (84.6%)	2076 (92.3%)	0.8 (0.7–1.1)	0.13
Electrolytes, urea, creatinine	1249 (83.8%)	2073 (92.1%)	0.8 (0.6–1.0)	0.05
C‐reactive protein	761 (51.0%)	1148 (51.0%)	1.1 (0.9–1.2)	0.97
Liver function test	730 (49.0%)	1170 (52.0%)	1.0 (0.9–1.2)	0.69
Blood gas test	699 (46.9%)	985 (43.8%)	1.3 (1.1–1.5)	< 0.001
**Microbiology**				
Blood culture	937 (62.8%)	1259 (56.0%)	1.3 (1.2–1.5)	< 0.001
Urine microscopy, culture and sensitivity analysis	392 (26.3%)	558 (24.8%)	1.0 (0.9–1.2)	0.17
Sputum culture	183 (12.3%)	192 (8.5%)	1.8 (1.4–2.3)	< 0.001
Respiratory bacteria serology	183 (12.3%)	164 (7.3%)	2.0 (1.6–2.6)	< 0.001
Respiratory virus serology	126 (8.5%)	119 (5.3%)	2.1 (1.6–2.8)	< 0.001

CI = confidence interval. ^*^ Standard *v* rapid PCR testing, adjusted for baseline characteristics (Box 1). ◆

### Sensitivity analyses

Of the 452 patients under 18 years of age in the standard PCR group, 301 (66.6%; 95% CI, 62.0–70.9%) were admitted to hospital, as were 158 of the 312 children (50.6%; 95% CI, 44.9–56.3%) in the rapid PCR group (standard *v* rapid PCR: aOR, 1.7; 95% CI, 1.3–2.4). Of the 670 patients over 60 years of age in the standard PCR group, 615 (91.8%; 95%, 89.4–93.8%) were admitted to hospital, as were 1167 of the 1384 patients of this age (84.3%; 95% CI, 82.3–86.2%) in the rapid PCR group (standard *v* rapid PCR: aOR, 2.1; 95% CI, 1.5–2.9) (Supporting Information, figure 1).

ED LOS was similar for the standard PCR and rapid PCR groups in both age‐based subgroups (Supporting Information, figure 2A). The differences in test turnaround time identified in the main analysis were also evident for each age‐based subgroup (Supporting Information, figure 2B).

## Discussion

In this before‐and‐after study, we found that rapid PCR testing of ED patients for major respiratory viruses was associated with significantly fewer admissions to hospital, more rapid delivery of test results, more patients receiving their test results before leaving the ED, and less frequent ordering of some common microbiology tests.

Other studies have also reported that hospital admission numbers were significantly lower when rapid influenza virus testing was used in EDs. An analysis of outcomes for more than 300 adults at a tertiary care centre in New York found that early diagnosis of respiratory infections was associated with significantly fewer hospitalisations of influenza‐positive patients.^7^ In a small Irish study (73 patients), the hospital admission rate for obstetric patients declined from 88% to 45% after on‐site rapid influenza PCR testing was introduced.^10^ The differences in clinical setting and patient group may explain the smaller decline in our study (from 78% to 67%). Non‐PCR‐based rapid diagnostic tests for respiratory viruses have also been associated with lower hospital admission rates.^11^, ^12^

The main reason for fewer hospital admissions of patients tested by rapid PCR may be that the earlier availability of results enables clinicians to quickly diagnose or exclude respiratory infections and to make timely and informed decisions about whether to discharge the patient or admit them to hospital. When standard PCR was used, in contrast, our findings suggest that these decisions were made before the test results were available. The possible benefits of not admitting patients to hospital, beyond those for individual patient management, include better infection control and outbreak prevention, as well as reduced demands on hospital resources.^13^, ^14^ The impact of rapid PCR testing on outbreak prevention and infection control measures should be evaluated. Rapid influenza virus testing may also have practical implications for hospital bed management.^10^, ^15^

ED LOS was similar in our study before and after the introduction of rapid PCR methods. This finding was not unexpected, as test turnaround time is not the only rate‐limiting factor for decision making in EDs.^16^ Before rapid PCR methods were introduced, the long turnaround time of multiplex PCR did not necessarily extend a patient's stay in the ED, as they were usually admitted to hospital or discharged home before the results were available. Consequently, more rapid delivery of test results alone would not reduce ED LOS.

Reports on the effect of rapid influenza virus testing and LOS have been conflicting. While evidence for an association between rapid testing and shorter overall inpatient LOS has been reported,^6^, ^11^ findings regarding ED LOS are inconsistent.^7^, ^17^, ^18^ For example, a Cochrane review based on three randomised controlled trials did not find a statistically significant association of rapid viral diagnosis with lower mean ED LOS.^18^ In a study in children, ED LOS was actually 26 minutes longer with rapid PCR; inpatient LOS did not differ between the two groups, but was significantly shorter when the analysis was limited to patients with positive test results.^6^

We found that ordering of some other microbiology tests, including blood culture, sputum culture, and respiratory bacterial and virus serology, was significantly less frequent for patients tested by rapid PCR. The effect of PCR‐based rapid testing on the ordering of other laboratory tests has not previously been reported, although some studies of antigen‐based point‐of‐care testing have examined this outcome.^12^ Consistent with our finding, several investigators have reported fewer blood culture tests^19^, ^20^ and basic biochemistry and haematology tests, including full blood count,^20^, ^21^ C‐reactive protein testing,^21^ and urinalysis,^20^, ^21^ when point‐of‐care testing was used.

The higher rate of positive results for patients tested by rapid PCR than for those tested by standard PCR may reflect a higher prevalence of influenza during 2017 than in 2016.

The rapid PCR system in our study accurately detects influenza viruses A/B and RSV but, unlike the standard multiplex PCR, cannot detect other clinically relevant respiratory viruses, such as rhinovirus, coronavirus, human metapneumovirus, parainfluenza virus, adenovirus, enterovirus, and bocavirus. If infection with other respiratory viruses is suspected, patients may therefore need further investigations. Standard multiplex PCR can provide broader information, as it can detect multiple respiratory viruses in a single run, although the long turnaround time restricts its suitability for urgent clinical decision making. Improving the turnaround time of multiplex PCR analysis may achieve better outcomes.

### Strengths and limitations

The strengths of our study include its relatively large sample size; further, unlike many similar investigations, ours was a multicentre study in four hospital EDs, enhancing the generalisability of our findings. However, our analyses were not adjusted for comorbid conditions, as this information was not available. Because our study was an uncontrolled before‐and‐after study, our results cannot be interpreted as indicating causal relationships. As with all non‐randomised trials, we could not fully account for all potential confounding variables, nor for temporal trends and other unmeasured factors, such as changes in clinician testing practices or differences in the prevalence and severity of disease during the two study periods.^22^ For example, a shortage of inpatient beds caused by a high prevalence of influenza could influence decisions in a busy ED about admitting patients to hospital. However, we attempted to reduce seasonal effects by selecting similar time frames for the two study periods, to reduce selection bias by including all ED patients tested for influenza virus and RSV, and to control for differences in baseline patient characteristics by applying multivariate modelling. As medications data were not available to us, we were unable to assess the impact of rapid PCR testing on antibiotic and antiviral drug use. Similarly, the cost–benefit balance of rapid testing was not evaluated because relevant data were not available. The cost per patient of rapid PCR testing is generally higher than for central laboratory testing, but our findings suggest potential savings through lower numbers of hospital admissions and reduced resource use. This question could be evaluated in a further study.

### Conclusion

Rapid PCR testing for influenza virus and RSV infections in patients attending EDs was associated with significant improvements in a range of patient and laboratory outcomes, suggesting potential benefits for both the patients and the health care system. A cost–benefit analysis could examine the impact of rapid PCR testing on bed management and antimicrobial drug prescribing.

## Competing interests

No relevant disclosures.
