# Supplementary material for: The impact of rapid molecular diagnostic testing for respiratory viruses on outcomes for emergency department patients
Source: Med J Aust. 2019 Mar 5;210(7):316–20. doi: 10.5694/mja2.50049 (PMC6617970; doi:10.5694/mja2.50049)
Supplement: Supplementary file 1 [file MJA2-210-316-s001.pdf]

The Medical Journal of Australia

# Supporting Information

## Supplementary graphs

**This appendix was part of the submitted manuscript and has been peer reviewed. It is posted as supplied by the authors.**

Appendix to: Wabe N, Li L, Lindeman R, et al. The impact of rapid molecular diagnostic testing for respiratory viruses on outcomes for emergency department patients. *Med J Aust* 2019; doi: 10.5694/mja2.50049.

**Figure 1. Proportions of patients admitted to hospital (with 95% confidence intervals), by age group**

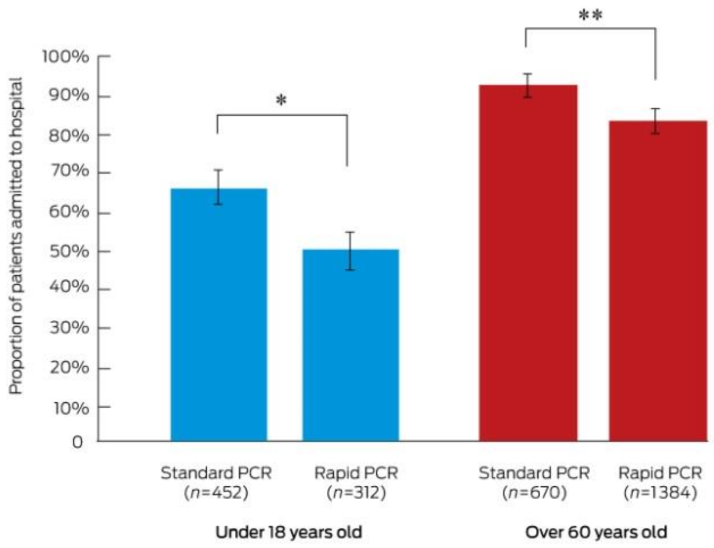


After adjusting for baseline characteristics (Box 1): * $P = 0.004$; ** $P < 0.001$.

**Figure 2. Emergency department lengths of stay (A), and test turnaround times (B) for patients, by age group**

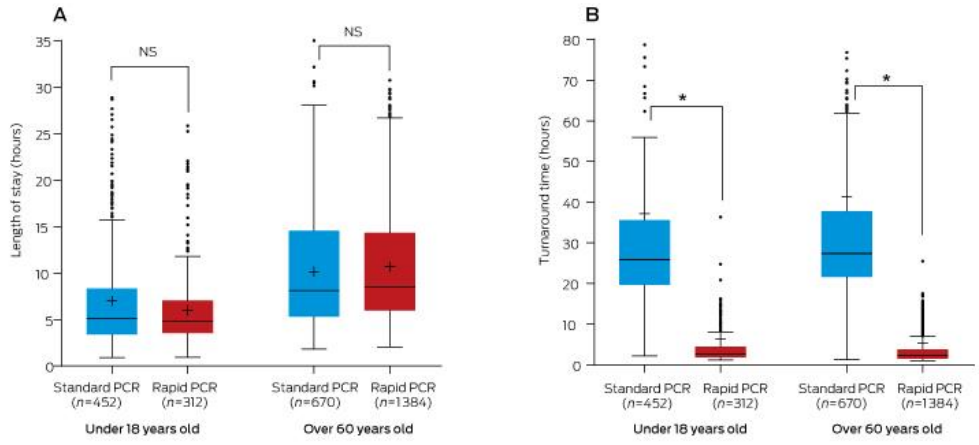


The box plots depict the median values and interquartile ranges (boxes), the 10th and 90th percentile values (whiskers), and outliers (dots). After adjusting for baseline characteristics (Box 1): NS = non-significant; * $P < 0.001$.
